# Supplementary material for: Photochemically-Induced Release of Lysosomal Sequestered Sunitinib: Obstacles for Therapeutic Efficacy
Source: Cancers (Basel). 2020 Feb 11;12(2):417. doi: 10.3390/cancers12020417 (PMC7072415; doi:10.3390/cancers12020417)
Supplement: Supplementary file 1 [file cancers-12-00417-s001.pdf]

Supplementary Materials: Photochemical-Induced Release of Lysosomal Sequestered Sunitinib: Obstacles for Therapeutic Efficacy

Judith Jing Wen Wong, Maria Brandal Berstad, Ane Sofie Viset Fremstedal, Kristian Berg, Sebastian Patzke, Vigdis Sørensen, Qian Peng, Pål Kristian Selbo and Anette Weyergang

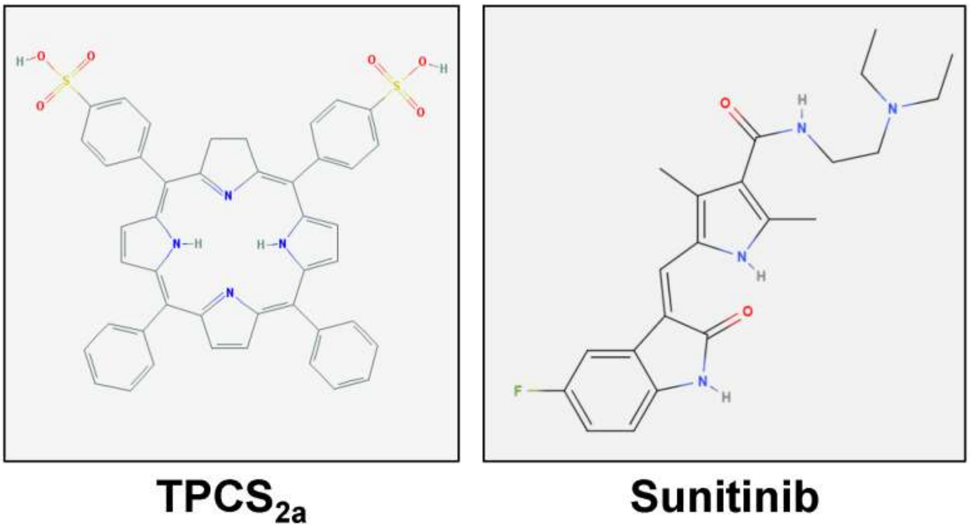

Figure S1. Chemical structure of disulfonated tetraphenyl chlorin ($TPCS_{2a}$) and sunitinib.

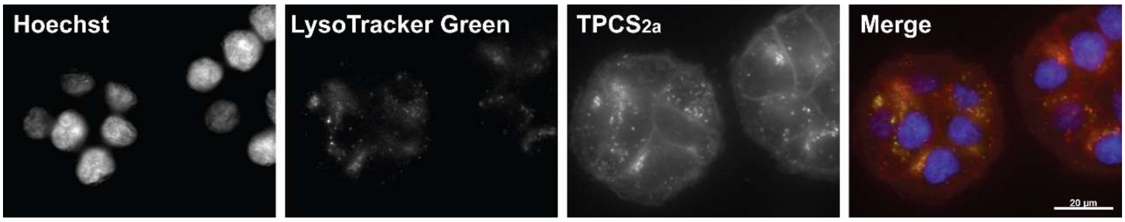

Figure S2. $TPCS_{2a}$ localization after 18 h incubation without wash. Representative live cell fluorescence imaging of $TPCS_{2a}$ in HT-29 cells after 18 h incubation with 0.4 µg/mL $TPCS_{2a}$ without wash and chase. $TPCS_{2a}$ (red), LysoTracker Green (green), Hoechst 33342 (blue). Co-localization indicated in yellow. Scale bar: 20 µm.

	Fluorescence (a.u.)	
**Sample**	**Alone**	**Combined**
Sunitinib	199.33 ± 11.08	106.55 ± 6.57
$TPCS_{2a}$	570.16 ± 6.51	460 ± 7.54

Figure S3. Signals from fluorescence spectroscopy of sunitinib, $TPCS_{2a}$ or the combination at pH~7 (PBS containing 1% FBS). Fluorescence detected in sunitinib, $TPCS_{2a}$ or the combination without light exposure. Data are mean of three experiments ± S.E.

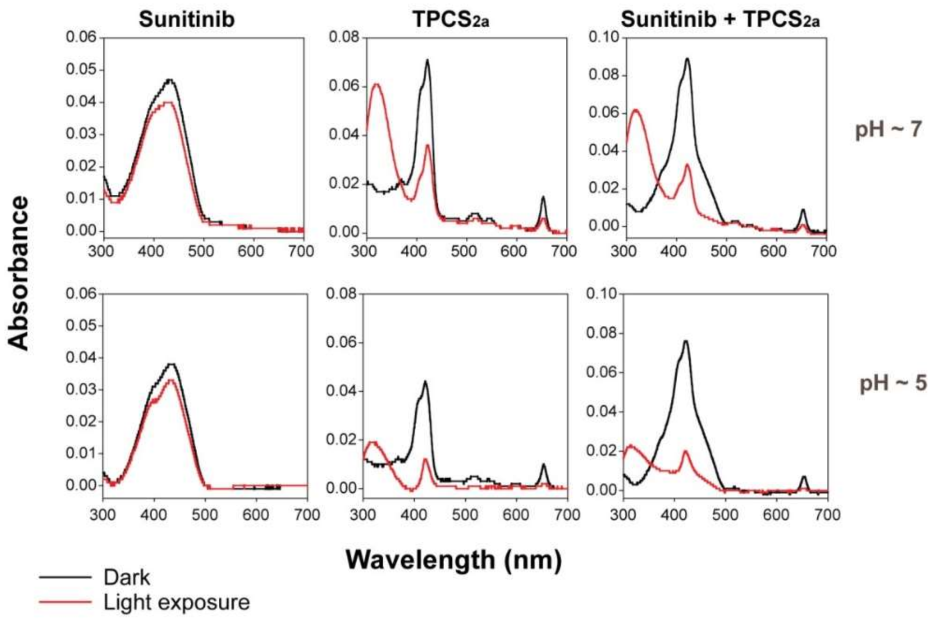

Figure S4. Absorbance spectra of sunitinib and $TPCS_{2a}$. Representative absorbance spectra of sunitinib alone, $TPCS_{2a}$ and the combination before and after blue light exposure at pH 7 and 5.

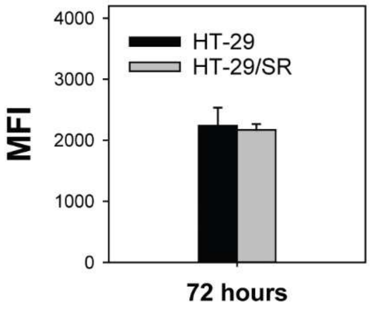

Figure S5. Sunitinib accumulation in HT-29 and HT-29/SR after 72 h incubation. Median sunitinib fluorescence intensities in live and single cells. Cells were subjected to a 24 h wash before incubation with sunitinib. (Mean of three experiments ± S.E.).

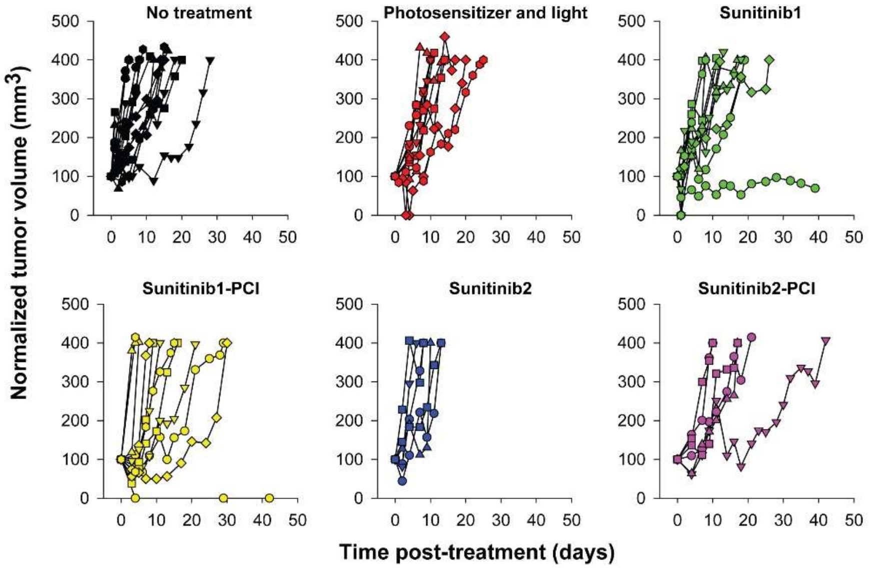

Figure S6. Tumor growth curves for HT-29 xenografts in athymic Nude-Foxn1^{nu} mice.

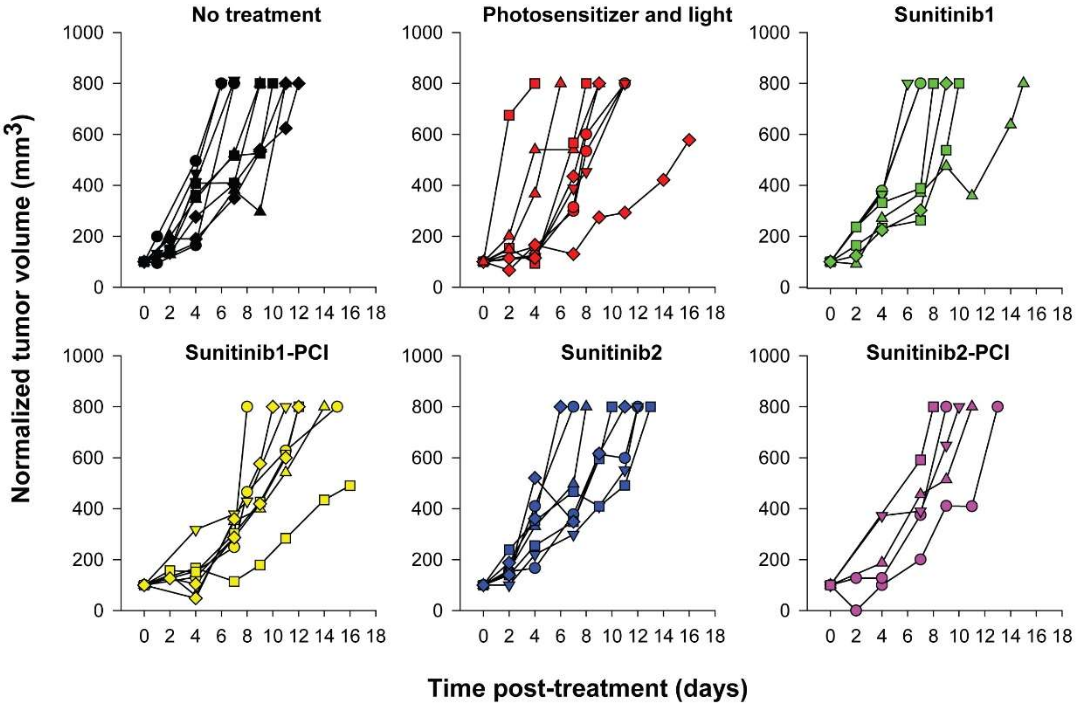

Figure S7. Tumor growth curves for CT26.WT allografts in BALB/c mice.

© 2020 by the authors. Licensee MDPI, Basel, Switzerland. This article is an open access article distributed under the terms and conditions of the Creative Commons Attribution (CC BY) license (http://creativecommons.org/licenses/by/4.0/).
